# *ELONGATED HYPOCOTYL5* ( *HY5* ) and *HY5 HOMOLOGUE* ( *HYH* ) maintain shade avoidance suppression in UV‐B

**DOI:** 10.1111/tpj.16328

**Published:** 2023-06-15

**Authors:** Ashutosh Sharma, Ashley J. Pridgeon, Wei Liu, Francisca Segers, Bhavana Sharma, Gareth I. Jenkins, Keara A. Franklin

**Affiliations:** ^1^ School of Biological Sciences, Life Sciences Building University of Bristol Bristol BS8 1TQ UK; ^2^ School of Molecular Biosciences, College of Medical, Veterinary and Life Sciences University of Glasgow Glasgow G12 8QQ UK

**Keywords:** shade avoidance, UV‐B, UVR8, HY5, HYH, *Arabidopsis thaliana*, gibberellin, DELLA

## Abstract

Reductions in red to far‐red ratio (R:FR) provide plants with an unambiguous signal of vegetational shade and are monitored by phytochrome photoreceptors. Plants integrate this information with other environmental cues to determine the proximity and density of encroaching vegetation. Shade‐sensitive species respond to reductions in R:FR by initiating a suite of developmental adaptations termed shade avoidance. These include the elongation of stems to facilitate light foraging. Hypocotyl elongation is driven by increased auxin biosynthesis promoted by PHYTOCHROME INTERACTING FACTORs (PIF) 4, 5 and 7. UV‐B perceived by the UV RESISTANCE LOCUS 8 (UVR8) photoreceptor rapidly inhibits shade avoidance, in part by suppressing *PIF4/5* transcript accumulation and destabilising PIF4/5 protein. Here, we show that longer‐term inhibition of shade avoidance is sustained by ELONGATED HYPOCOTYL 5 (HY5) and HY5 HOMOLOGUE (HYH), which regulate transcriptional reprogramming of genes involved in hormone signalling and cell wall modification. HY5 and HYH are elevated in UV‐B and suppress the expression of *XYLOGLUCAN ENDOTRANSGLUCOSYLASE/HYDROLASE* (*XTH*) genes involved in cell wall loosening. They additionally increase expression *GA2‐OXIDASE1* (*GA2ox1*) and *GA2ox2*, encoding gibberellin catabolism enzymes that act redundantly to stabilise the PIF‐inhibiting DELLA proteins. UVR8 therefore regulates temporally distinct signalling pathways to first rapidly inhibit and subsequently maintain suppression of shade avoidance following UV‐B exposure.

## Significance Statement

UV‐B perceived by the photoreceptor UVR8 provides a strong brake on plant shade avoidance responses. Following the rapid depletion of PHYTOCHROME INTERACTING FACTORS (PIFs), UV‐B‐mediated suppression of shade avoidance is maintained by the transcription factors HY5 and HYH, which regulate wide‐scale transcriptional reprogramming of genes involved in hormone signalling and cell wall loosening, stabilising PIF‐inhibiting DELLA proteins.

## INTRODUCTION

Shading by neighbouring vegetation presents a significant threat to the survival of shade‐intolerant species. Light quantity and quality signals are perceived by plant photoreceptors and integrated with information from mechanical stimulation and plant‐emitted volatiles to initiate an appropriate response (Pierik & de Wit, 2014). Light filtered through vegetation is depleted in red, blue and UV‐B light, and enriched in far‐red light. Reductions in red to far‐red ratio (R:FR) inactivate phyB, promoting shade avoidance responses. These include increased stem elongation and upwards leaf movement (hyponasty), elevating leaves towards sunlight before canopy closure. Low R:FR‐induced hypocotyl elongation is regulated by the bHLH transcription factors PHYTOCHROME INTERACTING FACTOR (PIF)4, PIF5 and PIF7, with PIF7 performing a dominant role (Fiorucci & Fankhauser, 2017). In high R:FR, the binding of phyB to PIF4 and PIF5 results in their phosphorylation, ubiquitination and degradation (Lorrain et al., 2008), while PIF7 is inactivated through phosphorylation (Li et al., 2012). PIF activity is further suppressed through the binding of multiple inhibitors to form inactive heterodimers (Hornitschek et al., 2009). These include the DELLA proteins that additionally promote PIF degradation (de Lucas et al., 2008; Li et al., 2016). In low R:FR, the inactivation of phyB stabilises PIF4 and PIF5 (Lorrain et al., 2008), while PIF7 becomes dephosphorylated and accumulates in the nucleus, where it regulates gene expression via chromatin remodelling (Huang et al., 2018; Li et al., 2012; Willige et al., 2021). PIFs increase auxin production in the cotyledons by elevating the expression of key auxin biosynthesis enzymes in the *TRYPTOPHAN AMINOTRANSFERASE OF ARABIDOPSIS1* (TAA1)‐YUCCA pathway (Hornitschek et al., 2012; Li et al., 2012). Auxin is then transported to the hypocotyl by PIN‐dependent polar auxin transport (Keuskamp et al., 2010). Low R:FR also promotes gibberellin (GA) signalling. This is achieved through enhanced expression of GA biosynthesis genes, and interaction between the double B‐box transcription factor BBX24 and DELLA proteins. Together, these processes result in the degradation and inhibition of DELLAs, alleviating PIF repression and promoting shade avoidance (Crocco et al., 2015; Devlin et al., 2003; Djakovic‐Petrovic et al., 2007).

In dense canopies, low R:FR signals are accompanied by low levels of blue and UV‐B light (Fraser et al., 2016). Resources are limited in true shade, so auxin sensitivity is enhanced through a mechanism requiring PIF4 and PIF5 (Hersch et al., 2014). Once a canopy gap is reached, UV‐B perceived by the UVR8 photoreceptor provides a clear sunlight signal that attenuates shade avoidance (Hayes et al., 2014; Mazza & Ballaré, 2015; Sharma et al., 2019; Tavridou, Pireyre, & Ulm, 2020; Tavridou, Schmid‐Siegert, et al., 2020). UVR8 exists as a dimer bound by salt‐bridge interactions. These are disrupted following UV‐B absorption by tryptophan residues, causing monomerisation. UVR8 monomers bind the CONSTITUTIVELY PHOTOMORPHOGENIC1/SUPPRESSOR OF PHYA‐105 (COP1/SPA) E3 ubiquitin ligase complex to initiate UV‐B signalling (Huang et al., 2013; Rizzini et al., 2011). Sequestration of COP1 by UVR8 destabilises PIF5, resulting in PIF5 protein degradation within 20 min of UV‐B perception (Sharma et al., 2019). UVR8‐mediated suppression of *PIF4* and *PIF5* transcript accumulation is also observed within 2–3 h (Hayes et al., 2014; Sharma et al., 2019; Tavridou, Schmid‐Siegert, et al., 2020). Together, these mechanisms provide a rapid mechanism to deplete PIFs and inhibit stem elongation.

Interaction between UVR8 and the COP1‐SPA complex also stabilises the basic leucine zipper transcription factors ELONGATED HYPOCOTYL5 (HY5) and HY5 HOMOLOGUE (HYH), which feed forward to activate their own transcription (Binkert et al., 2014; Favory et al., 2009; Huang et al., 2013). Mutants deficient in both HY5 and HYH display longer hypocotyls than wild‐type (WT) controls in continuous low R:FR + UV‐B, suggesting a role for HY5 and HYH in suppressing hypocotyl elongation in these conditions (Hayes et al., 2014). HY5/HYH were additionally shown to elevate *GA2ox1* expression in UV‐B, leading to speculation that this pathway may directly control DELLA abundance. The roles of UVR8, HY5/HYH and GA2ox enzymes in UV‐B‐mediated DELLA stabilisation were not, however, tested. In this study, we performed RNA sequencing analysis to identify HY5/HYH‐regulated genes involved in the UV‐B‐mediated inhibition of low R:FR‐induced shade avoidance. We found that HY5 and HYH regulate multiple genes involved in secondary metabolite production, including flavonoids, phenylpropanoids and diterpenoids; auxin signalling; GA signalling and cell wall expansion, including *GA2ox1*, *GA2ox2* and multiple *XTH* genes. We additionally performed a temporal analysis of UV‐B‐mediated DELLA stabilisation. This occurs later than PIF degradation and transcript suppression, requiring at least 4 h of UV‐B treatment. UV‐B‐mediated DELLA stabilisation involves UVR8, HY5/HYH and GA2ox enzymes, supporting the initial hypothesis of Hayes et al. (2014). Collectively we show that HY5/HYH are required for sustaining shade avoidance inhibition in UV‐B following rapid PIF depletion. This is achieved, in part, by suppressing GA signalling. Our results provide a refined, temporally phased model of how UV‐B signalling inhibits shade avoidance.

## RESULTS

### HY5 and HYH regulate UV‐B‐mediated transcriptional reprogramming of genes involved in hormone signalling and cell wall modification

**Figure 1:**
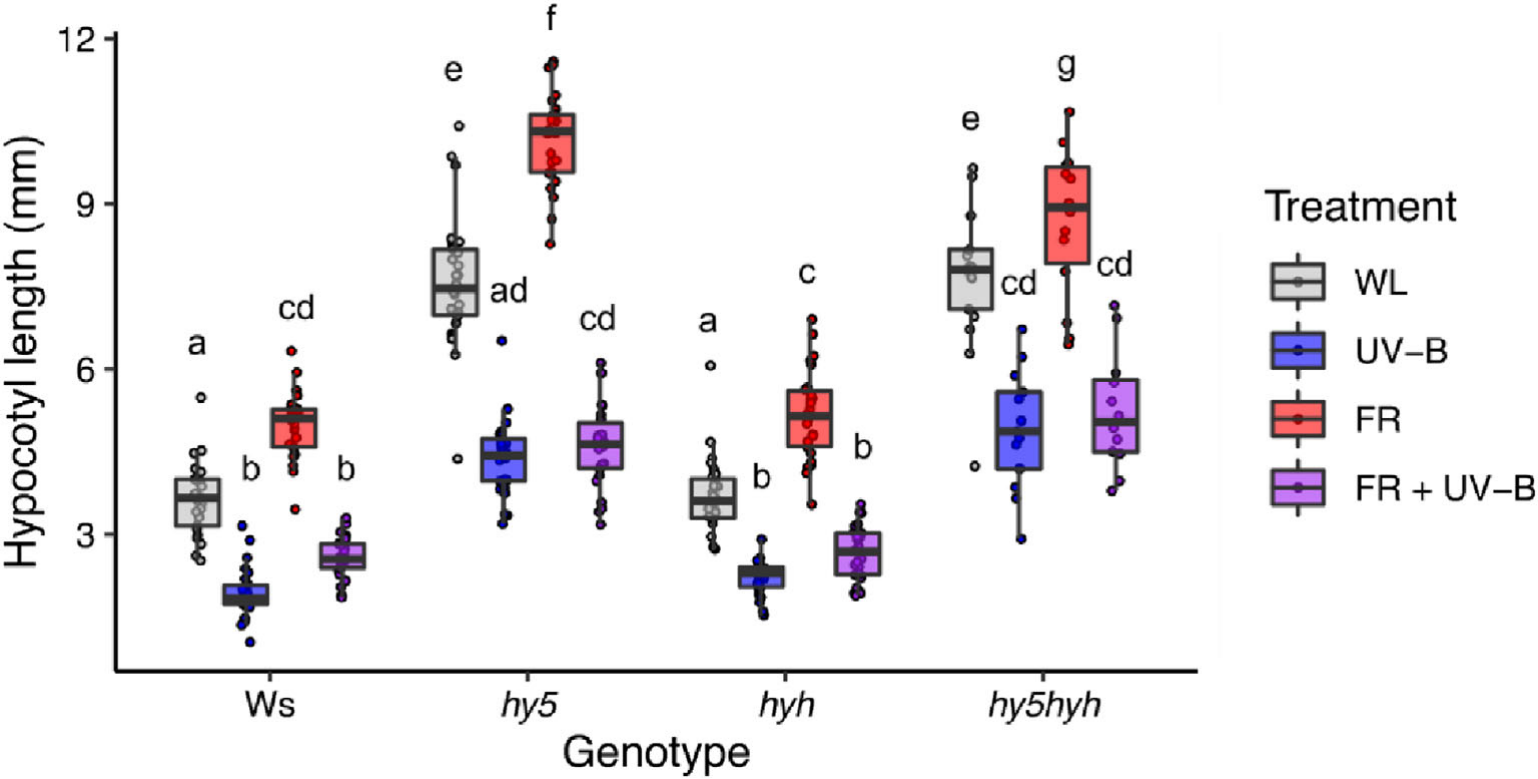
UV‐B‐mediated shade avoidance inhibition in 16 h light/8 h dark cycles involves HY5/HYH‐dependent and ‐independent processes. Hypocotyl lengths of wild‐type (WT) (Ws), *hy5*, *hyh* and *hy5/hyh* seedlings, grown in 16 h light, 8 h dark cycles at 20°C. Seedlings were grown for 3 days in white light (WL) before transfer to different light conditions (WL, + FR, + UV‐B or + FR + UV‐B) for 4 days. Red to far‐red ratio (R:FR) values were 6 (WL) or 0.07 (+ FR). UV‐B was provided at 1 μmol m^−2^ sec^−1^. Boxes represent 25th to 75th percentile. Bars show the median length of at least 12 individual seedlings, and whiskers represent spread of data within 1.5 * interquartile range. Different letters represent statistically different mean values (*P* < 0.05) using a two‐way ANOVA with Tukey multiple comparison test.

**Figure 2:**
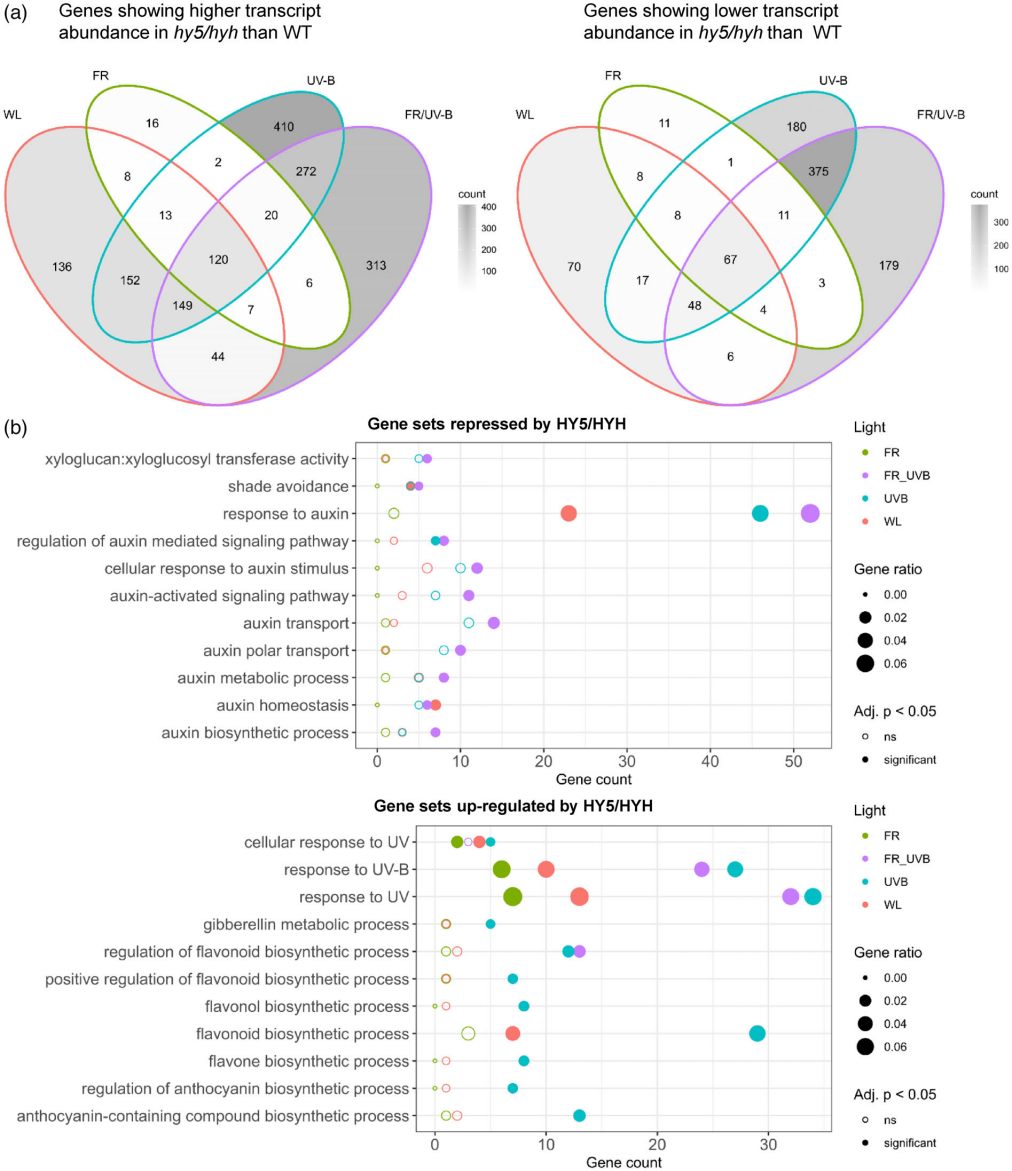
HY5/HYH regulate transcriptional reprogramming of genes involved in secondary metabolite production, hormone signalling and cell wall modification during UV‐B‐mediated inhibition of shade avoidance. (a) Venn diagrams showing overlap between differentially expressed gene sets (log2 fold‐change > |1| and adjusted *P* < 0.05) in different light treatments. Seedlings were grown for 7 days in white light (WL) at 20°C before transfer at dawn to different light conditions; WL, + FR (FR), + UV‐B (UV‐B) or + FR + UV‐B (FR/UV‐B) for 4 h. Red to far‐red ratio (R:FR) values were 8 (WL) or 0.06 (+ FR). UV‐B was provided at 1 μmol m^−2^ sec^−1^. (b) Gene ontology (GO) terms associated with light response, hormone signalling and cell wall modification among HY5/HYH‐regulated gene sets. Colours represent different light treatments, while a filled dot indicates that a GO term was found to be significantly enriched among the gene set (adjusted *P* < 0.05). The gene ratio was calculated by dividing the number of genes annotated with the respective GO term in the gene set by the total number of significantly differentially expressed genes with a GO annotation in the set. Note that some dots are overlapping.

The involvement of HY5 and HYH in UV‐B‐mediated inhibition of shade avoidance has been reported in continuous irradiation (Hayes et al., 2014; Tavridou, Pireyre, & Ulm, 2020). In 16‐h light/8‐h dark photocycles, similar results are obtained, with HY5 performing the dominant role (Figure 1). Consistent with published data in Col‐0 (Favory et al., 2009; Huang et al., 2013), UV‐B increased HY5 protein stability in Ws, although weak signals prevented accurate quantification of these data (Figure S1). To investigate the gene regulatory networks controlled by HY5/HYH during UV‐B‐mediated suppression of shade avoidance, RNA sequencing analysis was performed on Ws and *hy5/hyh* mutants treated with high and low R:FR ± UV‐B (Tables S1). Seedlings were grown in white light (WL) for 7 days before dawn transfer to high (8.0) or low (0.06) R:FR with and without UV‐B supplementation (1 μmol m^−2^ sec^−1^) for 4 h. HY5/HYH‐mediated changes in *GA2ox1* transcript abundance have been reported in similar light conditions at this time point (Hayes et al., 2014). The transcript abundance of 857 genes showed a greater than twofold change in transcript abundance between WT and *hy5/hyh* mutants in WL (Figures 2a and S2; Table S2). Of these, 629 genes displayed higher transcript abundance in the *hy5/hyh* mutant than WT controls, and 228 lower transcript abundance. In low R:FR (FR) conditions, 305 genes displayed HY5/HYH‐mediated regulation (Figures 2a and S2; Table S3). Of these, 192 genes displayed higher transcript abundance in the *hy5/hyh* mutant than WT controls, and 113 lower transcript abundance. In WL + UV‐B (UV‐B) and WL + FR + UV‐B (FRUV‐B), 1845 and 1624 genes were differentially regulated by HY5/HYH, respectively (Figures 2a and S2; Tables S4 and S5). In UV‐B, 1138 genes displayed higher transcript abundance in the *hy5/hyh* mutant than WT controls, and 707 lower transcript abundance (Figures 2a and S2; Table S4). In FRUV‐B, 931 genes displayed higher transcript abundance in the *hy5/hyh* mutant than WT controls, and 693 lower transcript abundance (Figures 2a and S2; Table S5). Considerable overlap was observed in HY5/HYH‐mediated gene regulation between the four light conditions, consistent with the central role of HY5/HYH in plant photomorphogenesis (Figure 2a; Gangappa & Botto, 2016).

The highest percentage of differently expressed genes between WT and *hy5/hyh* mutant plants was observed in environments containing UV‐B, reinforcing the importance of HY5 and HYH in UV‐B signalling (Figure 2a; Oravecz et al., 2006). Of these, gene ontology (GO) analysis showed HY5/HYH to strongly increase the transcript abundance of genes associated with UV signalling and secondary metabolite production in both high and low R:FR (Figure 2b; Table S6). These findings are supported by KEGG pathway analysis, which showed significant enrichment of pathways related to phenylpropanoid, flavonoid and flavonol biosynthesis in HY5/HYH‐regulated gene sets in UV‐B (Table S7). UVR8‐regulated marker genes *CHALCONE SYNTHASE* (*CHS*), *TRANSPARENT TESTA 7* (*TT7*), *SIGMA FACTOR 5* (*SIG5*) and *CRYPTOCHROME 3* (*CRY3*) all displayed HY5/HYH‐mediated transcript elevations in UV‐B, consistent with published studies (Table S4; Brown et al., 2005; Favory et al., 2009). A significant number of genes repressed by HY5/HYH in environments containing UV‐B were associated with auxin signalling and transport, consistent with the suppression of hypocotyl growth in these conditions (Figure S3; Table S6; Hayes et al., 2014; Tavridou, Pireyre, & Ulm, 2020). In contrast, genes involved in GA catabolism, associated with the GO term ‘gibberellin metabolic processes’, were positively regulated by HY5/HYH in UV‐B. Together, these data support a role for UVR8 and HY5/HYH signalling in suppressing the activity of growth‐promoting hormones (Hayes et al., 2014; Tavridou, Pireyre, & Ulm, 2020). The lowest number of HY5/HYH‐regulated genes was observed in low R:FR (Figure 2a). This aligns with published data showing a limited role for HY5/HYH in shade avoidance (Sellaro et al., 2011; van Gelderen et al., 2018).

**Figure 3:**
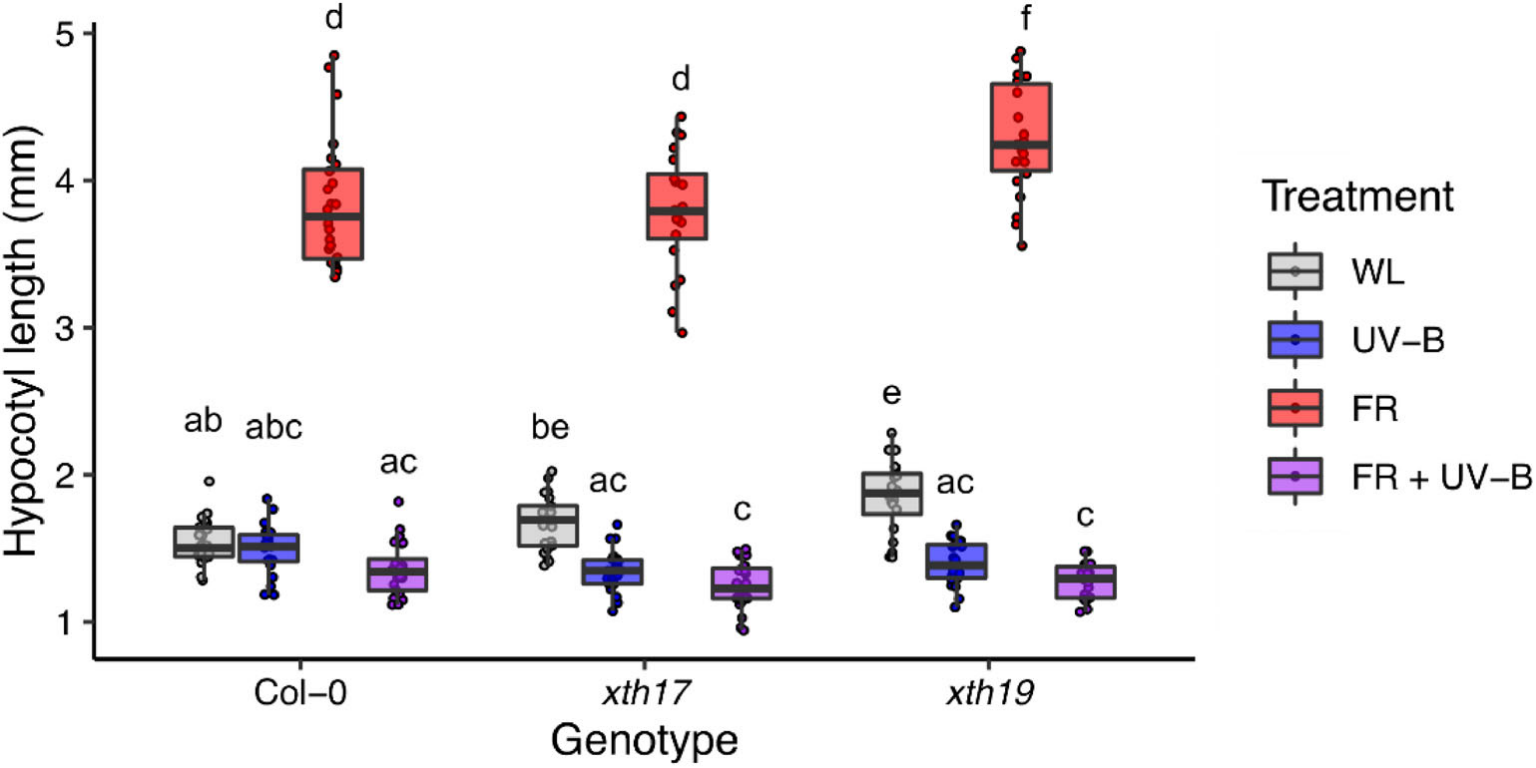
XTH enzymes are likely to act redundantly to control hypocotyl elongation during UV‐B‐mediated inhibition of shade avoidance. Hypocotyl lengths of Col, *xth17* and *xth19* seedlings grown in 16 h light/8 h dark cycles at 20°C. Seedlings were grown for 3 days in white light (WL) before transfer to different light conditions: WL, WL + UV‐B (UV‐B), WL + FR (FR) or WL + FR + UV‐B (FRUV‐B) for 4 days. Red to far‐red ratio (R:FR) values were 2.5 (WL) or 0.06 (FR). UV‐B was provided at 1 μmol m^−2^ sec^−1^. Boxes represent 25th to 75th percentile. Bars show the median, and whiskers represent spread of data within 1.5 * interquartile range (*n* ≥ 20). Different letters represent statistically different mean values (*P* < 0.05) using a two‐way ANOVA with Tukey multiple comparison test.

In addition to hormonal regulation, HY5/HYH were shown to negatively regulate gene sets associated with the GO term ‘xyloglucan:xyloglucosyl transferase activity’ in UV‐B. Xyloglucan is a polysaccharide essential for primary cell wall structure in dicotyledonous plants (Rose et al., 2002). Cell expansion requires extensive modification and loosening of the cellulose/xyloglucan network, and involves XYLOGLUCAN ENDOTRANSGLUCOSYLASE/HYDROLASE (XTH) enzymes (Van Sandt et al., 2007). The transcriptional regulation of some *XTH* genes has previously been shown to increase in low R:FR and decrease in UV‐B (Hornitschek et al., 2009; Sasidharan et al., 2010; Tavridou, Schmid‐Siegert, et al., 2020). Transcriptional suppression of *XTH* genes by HY5/HYH in UV‐B is therefore congruent with these findings (Hayes et al., 2014; Mazza & Ballaré, 2015; Sharma et al., 2019; Tavridou, Schmid‐Siegert, et al., 2020). In this study, *XTH17* and *XTH19* displayed significant HY5/HYH‐mediated suppression following 4 h of UV‐B treatment in low R:FR (Table S5). Similar results were obtained using quantitative polymerase chain reaction (qPCR), where UVR8‐mediated suppression of *XTH8* (Tavridou, Schmid‐Siegert, et al., 2020) was also observed (Figure S3). *xth17* and *xth19* single mutants showed similar UV‐B‐mediated shade avoidance inhibition to WT controls in both hypocotyls and petioles, suggesting redundancy between family members (Figures 3 and S4).

### *GA2ox* genes act redundantly to supress shade avoidance in UV‐B

**Figure 4:**
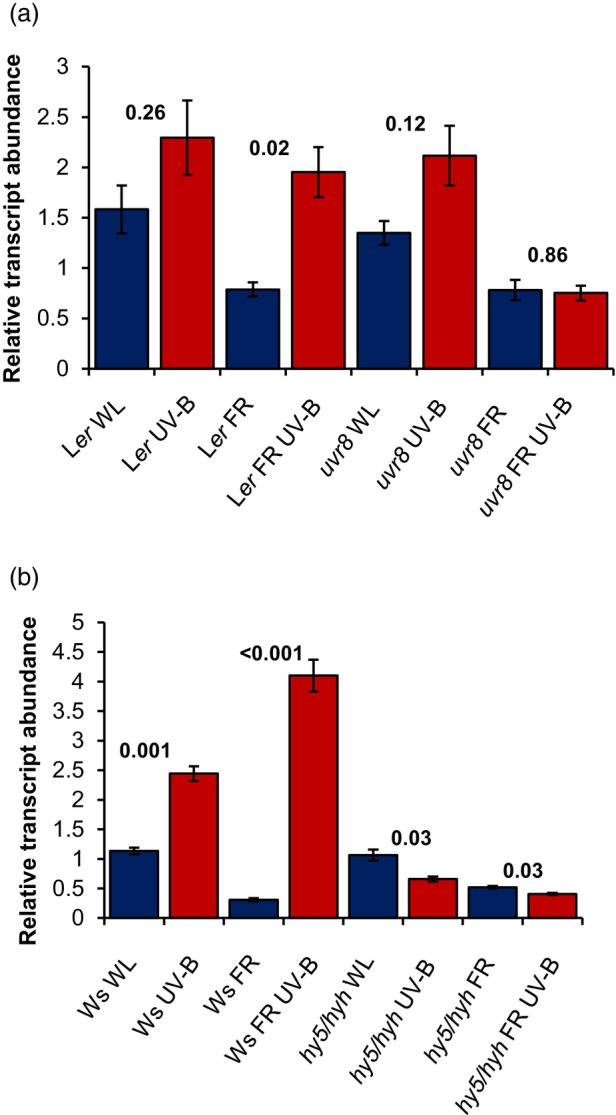
UV‐B perceived by UVR8 increases *GA2ox2* transcript abundance in a HY5/HYH‐dependent manner in low red to far‐red ratio (R:FR). Relative abundance of *GA2ox2* transcript in (a) L*er* and *uvr8‐1*, and (b) Ws and *hy5/hyh*. Seedlings were grown for 10 days in 16 h light/8 h dark cycles at 20°C before transfer at dawn to white light (WL), WL + UV‐B (UV‐B), WL + FR (FR) or WL + FR + UV‐B (FRUV‐B) for 4 h. R:FR values were 2.5 (WL) or 0.06 (+ FR). UV‐B was provided at 1 μmol m^−2^ sec^−1^. Transcript abundance was assayed by quantitative polymerase chain reaction (qPCR). Data are presented as the mean of three biological repeats (±) SE. *P*‐values from *t*‐tests comparing log‐transformed ΔΔCT values within genotypes are shown.

**Figure 5:**
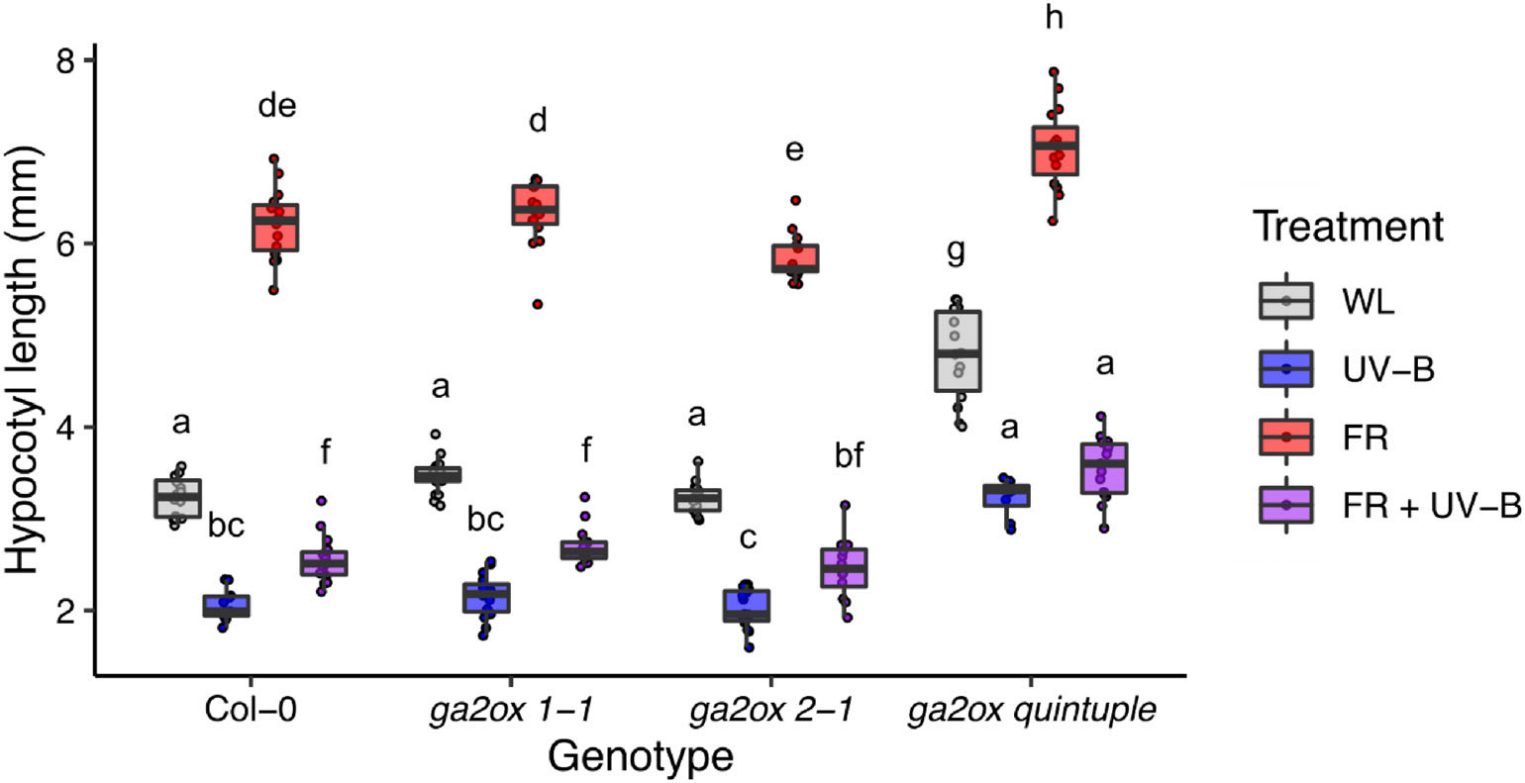
GA2oxidases act redundantly to control hypocotyl elongation in low red to far‐red ratio (R:FR) and UV‐B environments. Hypocotyl lengths of Col, *ga2ox 1‐1*, *ga2ox 2‐1* and *ga2ox* quintuple mutant seedlings grown in 16 h light/8 h dark photoperiods at 20°C. Seedlings were grown for 3 days in white light (WL) before transfer to different light conditions (WL, + FR, + UV‐B or + FR + UV‐B) for 4 days. R:FR values were 2.5 (WL) or 0.06 (+ FR). UV‐B was provided at 1 μmol m^−2^ sec^−1^. Boxes represent 25th to 75th percentile. Bars show the median lengths of at least 20 individual seedlings, and whiskers represent spread of data within 1.5 * interquartile range. Different letters represent statistically different mean values (*P* < 0.05) using a two‐way ANOVA with Tukey multiple comparison test.

UV‐B perceived by UVR8 increases *GA2ox1* transcript in a HY5/HYH‐dependent manner (Hayes et al., 2014). The role of GA2ox1 in DELLA stabilisation was not, however, tested in that study. GA2ox enzymes deactivate GAs by 2ß‐hydroxylation and are encoded by a family of five genes in *Arabidopsis*, *GA2ox1*, *GA2ox2*, *GA2ox3*, *GA2ox4* and *GA2ox6* (Hedden & Phillips, 2000). The roles of other *GA2ox* genes in UV‐B‐mediated suppression of shade avoidance have not been explored. Here, RNA‐seq analysis showed *GA2ox1* and *GA2ox2* transcripts to be elevated by UV‐B treatment in a HY5/HYH‐dependent manner (Tables S3 and S4). The roles of UVR8 and HY5/HYH in *GA2ox2* regulation were further confirmed by qPCR (Figure 4). In L*er*, a significant UVR8‐mediated increase in *GA2ox*2 transcript was observed in low R:FR (FRUV‐B) but not high R:FR (UV‐B). In Ws, a significant UV‐B‐mediated increase in *GA2ox2* transcript was observed in high and low R:FR. These increases were not observed in *hy5/hyh* mutants, confirming a role for HY5/HYH in this response (Figure 4). No UV‐B‐mediated increases in *GA2ox3*, *GA2ox4* or *GA2ox6* transcript were observed in RNA‐seq or qPCR studies (Tables S4 and S5; Figure S5). UV‐B perception by plants in this experiment was confirmed using *CHS* as a positive control (Figure S5). The importance of GA2ox1 and GA2ox2 in supressing shade avoidance in UV‐B was investigated by assaying seedling hypocotyl lengths of single and higher order *ga2ox* mutants in high and low R:FR ± UV‐B. *ga2ox1* and *ga2ox2* mutants resembled WT controls in all light conditions. The *ga2ox* quintuple mutant, deficient in all five GA2ox enzymes, displayed significantly longer hypocotyls than WT controls in all light conditions, with UV‐B treatment unable to suppress hypocotyl elongation to WT levels. The partial suppression of shade avoidance by UV‐B observed in *ga2ox* quintuple mutants suggests redundancy between GA2ox enzymes and redundancy between GA2ox‐dependent and ‐independent mechanisms in mediating this response (Figure 5).

### UVR8, HY5/HYH and GA2ox enzymes are required for DELLA stabilisation in UV‐B

**Figure 6:**
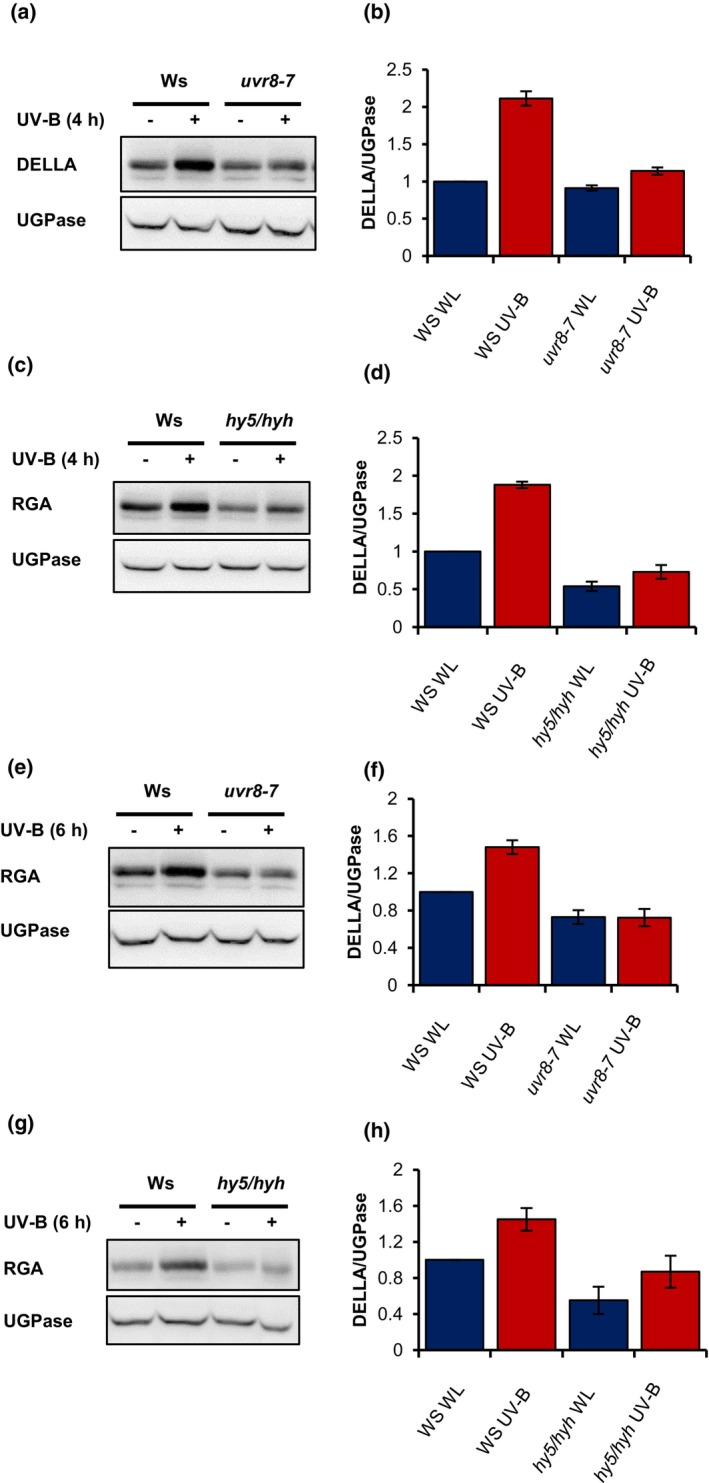
UV‐B‐mediated DELLA stabilisation involves UVR8 and HY5/HYH. Western blots of the DELLA protein, RGA and a UGPase loading control in Ws and *uvr8‐7* (a,e), and Ws and *hy5/hyh* (c,g). Seedlings were grown for 10 days in 16‐h light/8‐h dark cycles at 20°C before transfer at dawn to white light (WL) ± UV‐B (1 μmol m^−2^ sec^−1^) for 4 h (a–d) or 6 h (e–h). Mean DELLA/UGPase ratios are shown from two–four independent biological repeats (b,d,f,h). *n* = 2 (b,f) and *n* = 3 (d,h). Bars represent SE.

DELLA proteins are central repressors of plant growth. In addition to inhibiting PIF function (de Lucas et al., 2008; Li et al., 2016), they bind to nuclear prefoldin complexes to inhibit the polymerisation of cortical microtubules essential for anisotropic cell expansion (Locascio et al., 2013). Previous work has shown that a 6‐h treatment of low‐dose UV‐B can stabilise the DELLA protein RGA in both high and low R:FR, thereby contributing to the UV‐B‐mediated suppression of shade avoidance (Hayes et al., 2014). We therefore wished to determine the temporal regulation of this response and involvement of UVR8, HY5/HYH and GA2ox enzymes. No UV‐B‐, UVR8‐ or HY5/HYH‐mediated increase in DELLA (RGA) stability was observed following 1 h or 2 h of UV‐B treatment (Figure S6). UV‐B‐mediated DELLA stabilisation was, however, observed following 4 h and 6 h of treatment in a response requiring both UVR8 and HY5/HYH (Figure 6). This persisted following 12 h of UV‐B treatment in Ws, but not Col‐0 seedlings (Figure S7). Our earlier observations showed UV‐B to increase transcript abundance of the GA catabolising enzyme genes *GA2ox1* and *GA2ox2* (Figure 4; Tables S4 and S5) and GA2ox enzymes to redundantly regulate hypocotyl elongation in low R:FR and UV‐B (Figure 5). This led us to hypothesise that UVR8/HY5/HYH‐mediated elevations in GA2ox activity may directly stabilise DELLAs in UV‐B through reducing GA.

**Figure 7:**
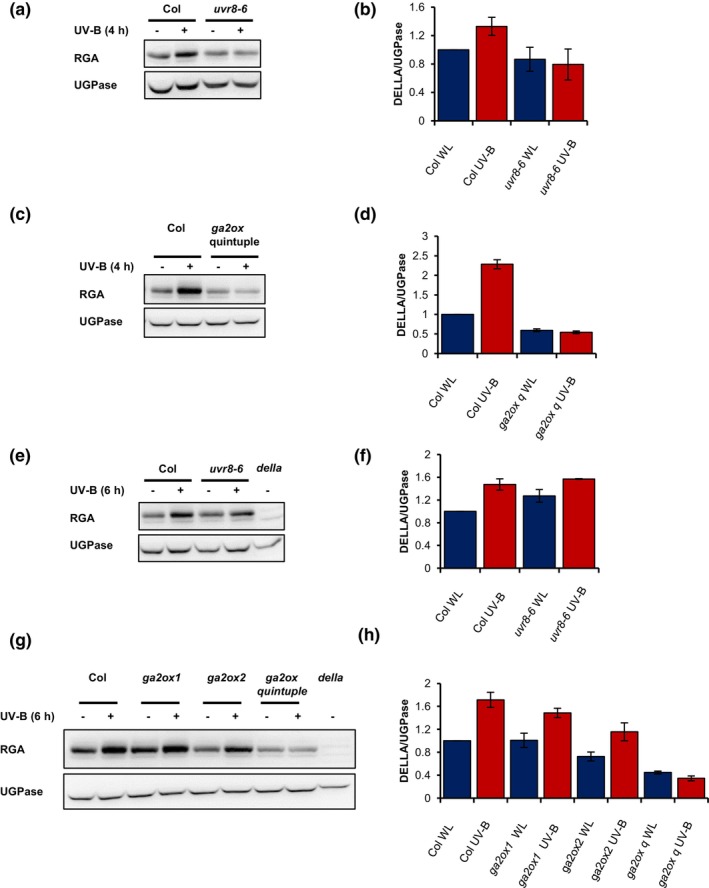
GA2oxidases act redundantly to enhance DELLA stability in UV‐B. Western blots of the DELLA protein, RGA and a UGPase loading control in Col, *uvr8‐6*, a DELLA quintuple null mutant (*della*), *ga2ox1*, *ga2ox2* and the *ga2ox* quintuple mutant (*ga2oxq*). Seedlings were grown for 10 days in 16‐h light/8‐h dark cycles at 20°C before transfer at dawn to white light (WL) ± UV‐B (1 μmol m^−2^ sec^−1^) for 4 h (a–d) or 6 h (e–h). Mean DELLA/UGPase ratios are shown from two–four independent biological repeats (b,d,f,h). *n* = 2 (b,f) and *n* = 4 (d,h). Bars represent SE.

To test this hypothesis, we quantified DELLA abundance in WT and *ga2ox* mutants in the presence and absence of UV‐B. The involvement of UVR8 was tested in parallel. At 4 h, UV‐B‐mediated DELLA stabilisation was absent in *uvr8* and *ga2ox* quintuple mutants, supporting the role of UVR8 and GA2oxidase enzymes in this response (Figure 7a–d). At 6 h, a reduced UV‐B‐mediated DELLA stabilisation response was observed in the *uvr8* mutant (Figure 7e,f). Mutants deficient in GA2ox2 displayed lower DELLA abundance than mutants deficient in GA2ox1, suggesting a greater role for the former in mediating DELLA stability. Mutants deficient in all five GA2ox enzymes displayed no UV‐B‐mediated DELLA stabilisation, suggesting this response to be mediated redundantly by multiple family members (Figure 7g,h).

## DISCUSSION

UV‐B signals the presence of sunlight to shaded plants emerging from canopy cover, limiting shade avoidance responses once a more favourable light environment has been reached (Hayes et al., 2014; Moriconi et al., 2018). In field experiments, the effectiveness of UV‐B in suppressing shade avoidance varies depending on both plant species and light conditions, but a clear UVR8‐dependent response has been observed (Mazza & Ballaré, 2015). UV‐B perceived by UVR8 suppresses auxin biosynthesis and stem elongation through a response involving both HY5/HYH‐dependent and ‐independent mechanisms (Hayes et al., 2014; Tavridou, Pireyre, & Ulm, 2020). HY5/HYH‐independent shade avoidance inhibition is likely to involve rapid UV‐B‐mediated degradation of PIF4 and PIF5 proteins (Hayes et al., 2014; Tavridou, Pireyre, & Ulm, 2020). Temporal analysis of UVR8‐mediated PIF5 degradation in low R:FR showed significant differences within 20 min (Sharma et al., 2019). This response required the N‐terminus of PIF5, and involved both COP1 and the ubiquitin‐proteosome. The authors propose that UVR8‐mediated sequestration of COP1 destabilises PIF5, possibly by exposing phyB binding sites on the PIF5 protein (Sharma et al., 2019). UVR8 additionally suppresses *PIF4* and *PIF5* transcript abundance over a longer timescale of 2–3 h (Hayes et al., 2014, 2017; Sharma et al., 2019; Tavridou, Schmid‐Siegert, et al., 2020), and inhibits PIF4/5 activity through stabilisation of the PIF‐binding bHLH transcription factor LONG HYPOCOTYL IN FAR RED 1 (HFR1) (Tavridou, Schmid‐Siegert, et al., 2020).

HY5 performs a wide variety of roles in plant environmental adaptation; integrating light, hormone and abiotic stress signalling pathways, while systemically coordinating shoot and root development to balance carbon and nitrogen acquisition (Chen et al., 2016; Gangappa & Botto, 2016; van Gelderen et al., 2018). This multifaceted role is supported by chromatin immunoprecipitation studies showing HY5 to bind ~3000 promoter targets in *Arabidopsis* (Lee et al., 2007). Forward genetic screens identified HY5 as a major component of UVR8 signalling, with its homologue, HYH, performing an accessory role (Brown Brown et al., 2005; Favory et al., 2009; Brown & Jenkins, 2008). In this study, an RNA sequencing approach was used to identify regulatory targets of HY5/HYH in UV‐B‐mediated shade avoidance inhibition. In low R:FR, 305 genes were differentially regulated by HY5/HYH, suggesting some role for these proteins in supressing shade avoidance in the absence of UV‐B (Figure 2). These data align with observations showing *hy5/hyh* mutants to display greater hypocotyl elongation than WT controls in both high and low R:FR (Figure 1; van Gelderen et al., 2018). Furthermore, *HY5* and *HYH* transcripts have been observed to increase 4–8 h after transfer to true shade (low R:FR, low PAR) in a response regulated by phyA, supporting their role in shade avoidance inhibition (Ciolfi et al., 2013). Here, the greatest number of HY5/HYH‐regulated genes was identified in environments containing UV‐B. Of these, the GO term ‘xyloglucan:xyloglucosyl transferase activity’ was associated with gene sets displaying decreased transcript abundance following UV‐B treatment in both high and low R:FR (Figure 2). We focussed our attention on XTH family as these enzymes have a key role in cell expansion, and *XTH* transcripts have previously been shown to increase in response to low R:FR (Rose et al., 2002; Hornitschek et al., 2009; Sasidharan et al., 2010; Tavridou, Schmid‐Siegert, et al., 2020). In *Arabidopsis*, 33 open reading frames have been identified that potentially encode XTH enzymes (Rose et al., 2002). *XTH9*, *‐15* (formerly known as *XTR7*), *‐16* and ‐*19* transcripts all increase in petioles in response to low R:FR, with *XTH15*, *‐16*, *‐17* and ‐*19* increasing in response to true shade (Sasidharan et al., 2010). In seedlings, low R:FR has been shown to increase the transcript of *XTH8*, *‐15*, *‐19* and ‐*22* (Hornitschek et al., 2009; Tavridou, Schmid‐Siegert, et al., 2020), with UV‐B‐mediated suppression of all observed within 3 h (Tavridou, Schmid‐Siegert, et al., 2020). We did not observe a role for HY5/HYH in UV‐B‐mediated suppression of *XTH15* in our experimental conditions, which may be explained, in part, by observations showing a UVR8‐independent component to this response (Tavridou, Schmid‐Siegert, et al., 2020). In continuous high R:FR, UV‐B supplementation resulted in the suppression of *XTH4* and *XTH33* transcript accumulation (Liang et al., 2018), suggesting that length of treatment may be a variable determining the responsivity of different family members. In addition to temporal specificity, potential functional redundancy between XTH enzymes makes analysis of single mutants problematic (Figures 3 and S3). Higher order mutants would therefore be required to determine the roles of family members in UV‐B‐mediated shade avoidance inhibition.

In agreement with Hayes et al. (2014), RNA‐seq analysis showed a HY5/HYH‐dependent increase in *GA2ox1* transcript following 4 h of UV‐B treatment (Tables S4 and S5). We additionally observed elevated *GA2ox2* transcript levels at this time point, suggesting that HY5/HYH stabilisation by UVR8 elevates multiple enzymes involved in GA catabolism (Tables S4 and S5; Figure 4). Redundancy of action was observed between GA2ox family members in both the regulation of DELLA stability and hypocotyl growth inhibition, suggesting the involvement of multiple family members in UV‐B‐mediated suppression of shade avoidance (Figures 5 and 7). Quantification of GA levels in WT and *hy5/hyh* plants grown in these experimental conditions would, however, be needed to confirm this hypothesis. As COP1 has been shown to accumulate in the nucleus during simulated shade (Pacín et al., 2013) and destabilise DELLA proteins (Blanco‐Touriñán et al., 2020), it is also possible that UVR8‐mediated sequestration of COP1 directly promotes DELLA stability in UV‐B. UVR8‐mediated increases in DELLA stability did, however, occur over a longer timescale than PIF degradation, consistent with the requirement for HY5/HYH. This response likely serves to inhibit any remaining PIF activity in addition to suppressing the polymerisation of cortical microtubules, further limiting hypocotyl cell elongation (de Lucas et al., 2008; Li et al., 2016; Locascio et al., 2013).

**Figure 8:**
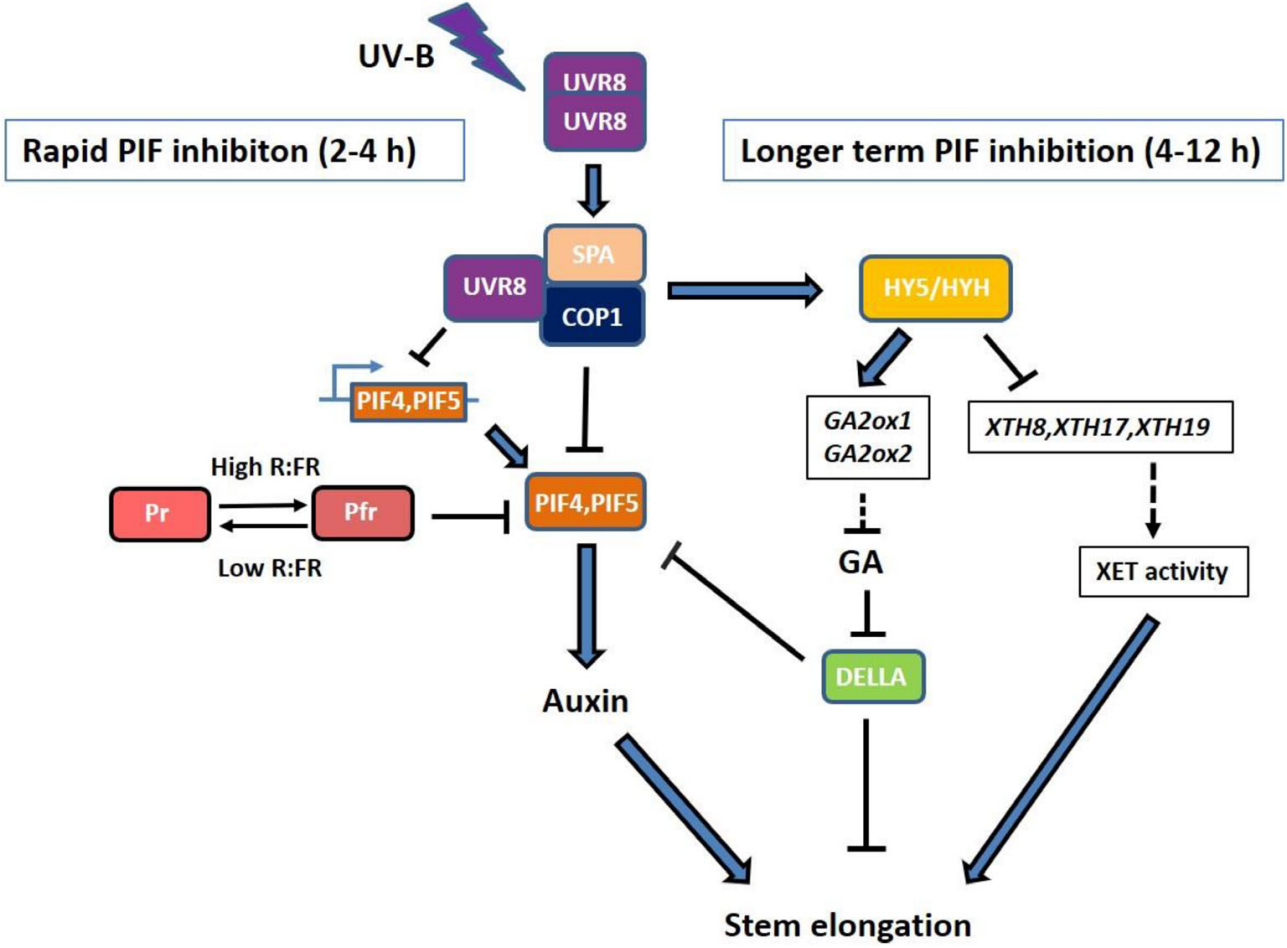
Hypothetical model showing how HY5/HYH contribute to longer term PIF inhibition and suppression of stem elongation in UV‐B. Low red to far‐red ratio (R:FR) promotes conversion of phytochrome B from the biologically active far‐red light‐absorbing (Pfr) form to the biologically inactive Pr form, stabilising PIF4 and PIF5. Perception of UV‐B drives monomerisation of UVR8, which forms a complex with COP1/SPA proteins. Sequestration of COP1/SPA leads to the rapid degradation of PIFs and suppression of *PIF4/5* transcript abundance. Over a longer timescale, COP1 sequestration by UVR8 additionally stabilises HY5/HYH, which act to supress the transcript abundance of *XTH* genes and promote the transcript abundance of two gibberellin (GA) catabolism genes, *GA2ox1* and *GA2ox2*. Suppression of *XTH* transcript accumulation is likely to inhibit XYLOGLUCAN ENDOTRANSGLUCOSYLASE (XET) activity, while the catabolism of GA by GA2oxidases leads to enhanced DELLA stability. DELLAs inhibit stem elongation through multiple mechanisms including the direct inhibition of PIF4/5 function.

Plant shade avoidance is regulated by a complex and dynamic signalling network, acting over multiple timescales (Ciolfi et al., 2013). In prolonged shade, shade avoidance responses are not maintained by elevated auxin levels, but by increased auxin sensitivity, achieved in part by increasing the abundance of auxin receptors (Pucciariello et al., 2018). In these conditions, PIF4 abundance is reduced in cotyledons, consistent with reduced auxin production, but maintained in hypocotyls, congruent with the hypothesised role of PIF4 and PIF5 in enhancing auxin sensitivity (Nozue et al., 2011; Pucciariello et al., 2018). Here we propose that following a rapid decrease in PIF abundance through sequestration of COP1 (Sharma et al., 2019), UVR8‐mediated stabilisation of HY5/HYH acts to maintain longer term suppression of PIF activity through repressing XTH activity and increasing GA catabolism. The latter would serve to stabilise DELLA proteins, alongside direct stabilisation resulting from UVR8‐mediated sequestration of COP1 (Figure 8). Multiple, temporally separated mechanisms of UV‐B‐mediated shade avoidance suppression may serve to provide plants with a rapidly reversible component in response to transient sunlight exposure and longer‐term reinforcement of the response in continued illumination. Although this study focusses on only one component of shade light (low R:FR), UV‐B perceived by UVR8 has additionally been shown to suppress low blue light‐mediated shade avoidance (Hayes et al., 2014), suggesting that this mechanism is likely to operate in true canopy shade. HY5 has been identified as central to long‐term shade avoidance suppression by daily sunflecks, even in the presence of UV‐B filters (Moriconi et al., 2018). It is likely that UVR8‐mediated increases in HY5 enhance this response in natural environments. By combining temporal analyses with multiple light treatments, a refined picture of plant responses to fluctuating light environments within canopies can be obtained.

## EXPERIMENTAL PROCEDURES

### Plant materials and growth conditions

Landsberg *erecta* (L*er*), Wassilewskija (Ws) and Columbia‐0 (Col) accessions of *Arabidopsis* were used as WT controls in this study. *hy5 Ks50*, *hyh* and *hy5Ks50hyh* mutants have been described elsewhere (Holm et al., 2002; Oyama et al., 1997). *xth17* (SALK_015077) and *xth19* (SALK_034274) mutants were kindly provided by Prof. Kazuhiko Nishitani (Tohoku University, Japan) and genotyped to select homozygous lines. Primers used in genotyping are shown in Table S8. Both mutants have been described in Osato et al. (2006). *ga2ox1‐1* and *ga2ox* quintuple mutants were kindly provided by Andrew Phillips (Rothamstead Research, UK). The *ga2ox2* mutant (SALK_051749) was obtained from the Nottingham Arabidopsis Stock Centre (NASC) and genotyped before use. All have been described elsewhere (Rieu et al., 2008). *Arabidopsis* seeds were sown directly onto a 3:1 mixture of compost and horticultural silver sand. Unless otherwise stated, seeds were stratified for 3 days in darkness at 4°C before transfer to controlled growth cabinets (Microclima 1600E, Snijder Scientific, The Netherlands) set to 16‐h light /8‐h dark cycles (R:FR ~ 2.5) at 20°C and 70% humidity. WL was provided by cool‐white fluorescent tubes (400–700 nm) at photon irradiance of 80 μmol m^−2^ sec^−1^. Supplementary narrowband UV‐B (~1.0 μmol m^−2^ sec^−1^) was provided by Philips TL100W/01 tubes. Supplementary FR LEDs positioned overhead (peak emission 735 nm) reduced R:FR to 0.06 for low R:FR experiments. All light measurements were performed using an Ocean Optics FLAME‐S‐UV–VIS spectrometer with a cosine corrector (oceanoptics.com).

### mRNA sequencing

Ws and *hy5/hyh* seedlings were grown for 7 days in continuous WL before transfer at dawn to WL, + FR, + UV‐B or + FR + UV‐B for 4 h. Aerial tissues of seedlings were harvested for RNA extraction from three independent biological repeats. Twenty‐four total RNA samples were supplied to the Bristol Genomics Facility (University of Bristol, UK) for library preparation and sequencing. A total yield of 250 ng of total RNA sample was taken directly into the Illumina TruSeq Stranded mRNA Library Preparation Kit (Illumina, San Diego, USA), and the protocol was followed according to manufacturer's instructions without deviation. Final libraries were quantified using the Thermofisher High Sensitivity dsDNA Qubit assay (Thermofisher Scientific, Waltham, MA, USA), and validated using the TapeStation (Agilent) DNA1000 screentape assay on the Agilent 2200 TapeStation instrument. The libraries were normalised to 4 nm, pooled equimolarly, and diluted to 1.6 pm for sequencing on the Illumina NextSeq500 instrument with NextSeq Control Software 2.4.6 using an Illumina NextSeq High Output Version 2, 150‐cycle sequencing kit (Illumina, San Diego, CA, USA) to generate 2 × 75‐bp paired reads. A spike‐in of a PhiX control library Version 3 (Illumina, San Diego, CA, USA) at 1% was added to the sequencing run to assist with accurate base calling and to check run quality. Primary data analysis was completed by on‐board software RTA Version 2.4.6.

### Bioinformatic analysis

An average of 50.28 million read pairs was obtained per sample (Table S1). The paired‐end 75‐bp reads were trimmed to remove low‐quality bases and any remaining adapter sequences with Fastp v0.23.2 (Chen et al., 2018). After trimming, on average 47.63 million read pairs per sample remained. For each sample, read quality before and after trimming was examined with FastQC v0.11.5 (Andrews, 2010). Reads were aligned against the *Arabidopsis thaliana* TAIR10 genome assembly downloaded from plants.ensembl.org (build GCA_000001735.1) using HISAT2 v2.2.1 (Kim et al., 2019). Gene count tables were generated with HTSeq‐count v0.13.5 (Anders et al., 2015). HTSeq‐count only considers those read pairs of which both sequences align to the reference genome, which was on average 45.10 million reads pairs per sample (Table S1).

To explore which genes are differentially expressed between Ws and *hy5/hyh* seedlings under different light treatments, pairwise comparisons were performed between the two genotypes for each of the four light conditions (WL, FR, UV‐B and FR/UV‐B). Differential gene expression analysis was performed using the R (v4.0.3, Core Team, 2021) package DESeq2 v1.30.1 (Love et al., 2014). *P*‐values were corrected with the ‘FDR’ method. Genes were considered to be differentially expressed if their log2 fold‐change was larger than 1 or smaller than −1, and the adjusted *P*‐value was smaller than 0.05. Plots showing the adjusted *P*‐values and log2 fold‐changes for each pairwise comparison were made with the R package EnhancedVolcano v1.8.0. To visualise overlap between differentially expressed gene sets of different comparisons, Venn diagrams were generated with the R package ggVennDiagram v1. Functional enrichment of GO terms among differentially expressed genes was tested using the *enrichGO* function of the R package clusterProfiler v3.18.1 (Yu et al., 2012). The TAIR GO annotations (org.At.tair.db: AH84114) were retrieved with AnnotationHub v2.22.1 for use within clusterProfiler. Tests for significantly enriched KEGG pathways among the differentially expressed gene sets were also performed on the PlantsGSEA platform (Yi et al., 2013), using the default parameters.

### Hypocotyl and petiole length measurement

Hypocotyl and petiole lengths were quantified using ImageJ software (rsb.info.nih.gov/ij/). For hypocotyl measurements, seedlings were grown for 3 days in 16‐h light/8‐h dark cycles of WL before transfer to WL, + FR, + UV‐B or + FR + UV‐B for a further 4 days. A minimum of 12 seedlings was measured for each genotype in each condition. For petiole length measurements, plants were grown in 16‐h light/8‐h dark cycles of WL for 10 days before transfer to WL, + FR, + UV‐B or + FR + UV‐B for a further 9 days. A minimum of seven plants was measured for each genotype in each condition. The largest fully expanded rosette leaf was used for petiole measurement. All experiments were repeated multiple times, with similar results.

### Quantitative real‐time PCR of transcript abundance

Seedlings were grown in 16‐h light/8‐h dark cycles at 20°C for 10 days, before transfer at dawn to WL, WL + UV‐B (UV‐B), WL + FR (FR) or WL + FR + UV‐B (FRUV‐B) for 4 h. Fifty micrograms of aerial tissue was harvested into liquid nitrogen and RNA extracted using a spectrum total RNA kit (Sigma, Burlington, MA, USA) according to the manufacturer's instructions. This was reverse transcribed using a High Capacity cDNA Reverse Transcription kit (Applied Biosystems, Waltham, MA, USA). Real‐time PCR reactions were performed with 2X Brilliant III SYBR Green QPCR (Agilent Technologies, Santa Clara, CA, USA) and data analysed using MxPro software (Agilent Technologies). Transcript levels were normalised to *ACTIN2*. Primer sequences are provided in Table S9.

### Protein extraction and immunoblotting

Seedlings were grown as described above for qPCR analyses. For RGA analyses, frozen samples were ground into fine power then mixed with extraction buffer [50 mm Tris‐HCl, pH 7.5, 150 mm NaCl, 1% Na deoxycholate, 0.5% (v/v) Triton X‐100, 1 mm DTT, 10 μl ml^−1^ Sigma protease inhibitor cocktail, 50 μm MG132]. After centrifugation at 14 000 ***g*** for 10 min at 4°C, proteins in supernatants were quantified using a Bradford assay (Bio‐Rad, Hercules, CA, USA). Forty micrograms of protein was mixed with sodium dodecyl sulphate–polyacrylamide gel electrophoresis (SDS–PAGE) sample buffer [4 × 250 mm Tris HCl, pH 6.8, 2% SDS, 40% (v/v) glycerol, 20% (v/v) β‐mercaptoethanol, 0.5% bromophenol blue] and heated for 5 min at 95°C before resolving on 8% SDS–PAGE gels. Proteins were transferred to PVDF membrane and visualised by staining with Ponceau S. Membranes were blocked in 10% skimmed milk powder in TBST (20 mm Tris‐HCl, pH 7.6, 150 mm NaCl, 1% Tween 20) for 1 h. For RGA detection, membranes were incubated in a 1:2000 dilution of anti‐RGA antibody (AS11 1630 Agrisera) overnight at 4°C before incubation in a 1:10 000 dilution of secondary anti‐rabbit antibody (Promega Cat No W4011, Madison, WI, USA). Signals were detected using SuperSignal West Femto maximum sensitivity substrate (Thermo Fisher) and visualised using a Fusion Pulse imager (Vilber Lourmat, Eberhardzell, Germany). For protein quantification, EvolutionCapt software was used to determine the density of bands on immunoblots, using two different exposure times with unsaturated signals. Background signals were subtracted and the average of two exposures used to determine protein density. Membranes were then stripped and re‐probed in a 1:5000 dilution of anti‐UGPase antibody (Agrisera) followed by a 1:20 000 dilution of anti‐rabbit antibody (Promega Cat No W4011) to provide a loading control. For detection of HY5, the methods used for total protein extraction from plant tissue, SDS–PAGE and immunodetection on Western blots were as described previously (Kaiserli & Jenkins, 2007; Liao et al., 2020). An anti‐HY5 antibody (Agrisera, Vännäs, Sweden) was used for immunodetection. A *uvr8‐1* mutant expressing GFP‐UVR8 (Cloix & Jenkins, 2008) was used as a positive control.

## AUTHOR CONTRIBUTIONS

AS, AJP, WL, GIJ and KF designed experiments. AS, AJP, WL and BS performed experiments. FS analysed RNAseq data. AS, AJP, WL, FS, GIJ and KAF prepared the manuscript. All authors provided feedback on the manuscript.

## CONFLICT OF INTEREST

The authors declare no conflict of interest.

## Supporting information


**Figure S1.** UV‐B increases HY5 abundance in Ws seedlings.
**Figure S2.** Volcano plots of differentially expressed transcripts in Ws and *hy5/hyh* seedlings treated with (a) WL, (b) WL + UV‐B, (c) WL + FR and (d) WL + FR + UV‐B.
**Figure S3.** UV‐B‐mediated suppression of *XTH* transcript abundance requires UVR8.
**Figure S4.** XTH enzymes are likely to act redundantly to control petiole elongation during shade avoidance.
**Figure S5.** UV‐B does not increase *GA2ox3*, *GA2ox4* or *GA2ox6* transcript abundance.
**Figure S6.** UVR8‐ and HY5/HYH‐mediated increases in DELLA stability do not occur within 2 h of UV‐B treatment.
**Figure S7.** UV‐B‐mediated increases in DELLA stability involving HY5/HYH and GA2oxidases are maintained at 12 h in Ws but not Col‐0.
**Table S8** Primers used for genotyping *xth* and *ga2ox2* mutant lines
**Table S9** Primer sequences used for qPCR
F = forward primer, R = reverse primer.

**Table S1** Table lists for each sample the number of reads before and after trimming with Fastp v0.23.2
The overall alignment rate represents the percentage of trimmed reads that could be mapped with HISAT2 v2.2.1 to the *A. thaliana* TAIR10 genome assembly downloaded from plants.ensembl.org.
Only pairs of which both reads mapped to the reference were counted with HTSeq‐count v0.13.5 (last column of table).
**Table S2** Genes that were significantly (adj. *P* < 0.05 and log2 fold‐change > |1|) differentially expressed between Ws and hy5/hyh seedlings under WL light conditions
**Table S3** Genes that were significantly (adj. *P* < 0.05 and log2 fold‐change > |1|) differentially expressed between Ws and hy5/hyh seedlings under FR light conditions
**Table S4** Genes that were significantly (adj. *P* < 0.05 and log2 fold‐change > |1|) differentially expressed between Ws and hy5/hyh seedlings under UV‐B light conditions
**Table S5** Genes that were significantly (adj. *P* < 0.05 and log2 fold‐change > |1|) differentially expressed between Ws and hy5/hyh seedlings under FR/UV‐B light conditions
**Table S6** Significantly enriched GO terms (Biological Process and Molecular Function) among the higher and lower expressed gene sets (hy5/hyh versus Ws) for each light condition (WL, FR, UV‐B, FR/UV‐B)
The *P*‐values were adjusted with the ‘FDR’ method as implemented in clusterProfiler
**Table S7** The significantly enriched KEGG pathways among the gene sets
The tests were performed on the PlantsGSEA platform (http://structuralbiology.cau.edu.cn/PlantGSEA/index.php), using the default testing parameters.
Higher or lower means higher or lower expressed in hy5/hyh compared with Ws seedlings under the given light conditions.

## Data Availability

The RNA sequencing data supporting this study can be retrieved from the GEO repository (accession GSE192469).
