# Supplementary material for: ELONGATED HYPOCOTYL5 ( HY5 ) and HY5 HOMOLOGUE ( HYH ) maintain shade avoidance suppression in UV‐B
Source: Plant J. 2023 Jun 15;115(5):1394–407. doi: 10.1111/tpj.16328 (PMC10953383; doi:10.1111/tpj.16328)
Supplement: Supplementary file 1 — Figure S1. UV‐B increases HY5 abundance in Ws seedlings. Figure S2. Volcano plots of differentially expressed transcripts in Ws and hy5/hyh seedlings treated with (a) WL, (b) WL + UV‐B, (c) WL + FR and (d) WL + FR + UV‐B. Figure S3. UV‐B‐mediated suppression of XTH transcript abundance requires UVR8. Figure S4. XTH enzymes are likely to act redundantly to control petiole elongation during shade avoidance. Figure S5. UV‐B does not increase GA2ox3, GA2ox4 or GA2ox6 transcript abundance. Figure S6. UVR8‐ and HY5/HYH‐mediated increases in DELLA stability do not occur within 2 h of UV‐B treatment. Figure S7. UV‐B‐mediated increases in DELLA stability involving HY5/HYH and GA2oxidases are maintained at 12 h in Ws but not Col‐0. Table S8 Primers used for genotyping xth and ga2ox2 mutant lines Table S9 Primer sequences used for qPCR F = forward primer, R = reverse primer. [file TPJ-115-1394-s002.pdf]

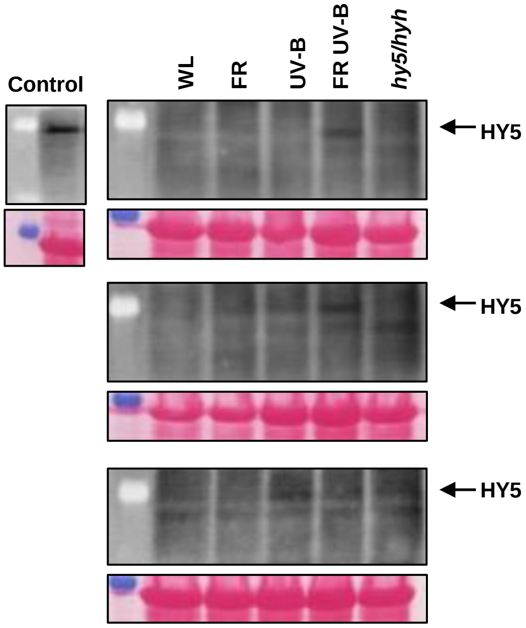

Figure S1. UV-B increases HY5 abundance in Ws seedlings. Western blots of HY5 in Ws seedlings grown for 5 d in 16 h light/ 8 h dark cycles at 20ºC before transfer at dawn to WL, WL+UV-B (UV-B), WL+FR (FR) or WL+FR+UV-B (FRUV-B) for 4 h. R:FR values were 2.5 (WL) or 0.06 (+FR). UV-B was provided at 1 μmol$m^{-2}s^{-1}$. *hy5/hyh* mutants (Ws) grown in WL were used as a negative control. A transgenic *uvr8-1*/*GFP-UVR8* line (L*er*) grown in WL was used as a positive control. 120 µg protein was loaded for Ws samples and 50 µg for the positive control. Ponceau staining of the Rubisco large subunit (rbcL) was used as loading control. Each blot represents an independent biological repeat.

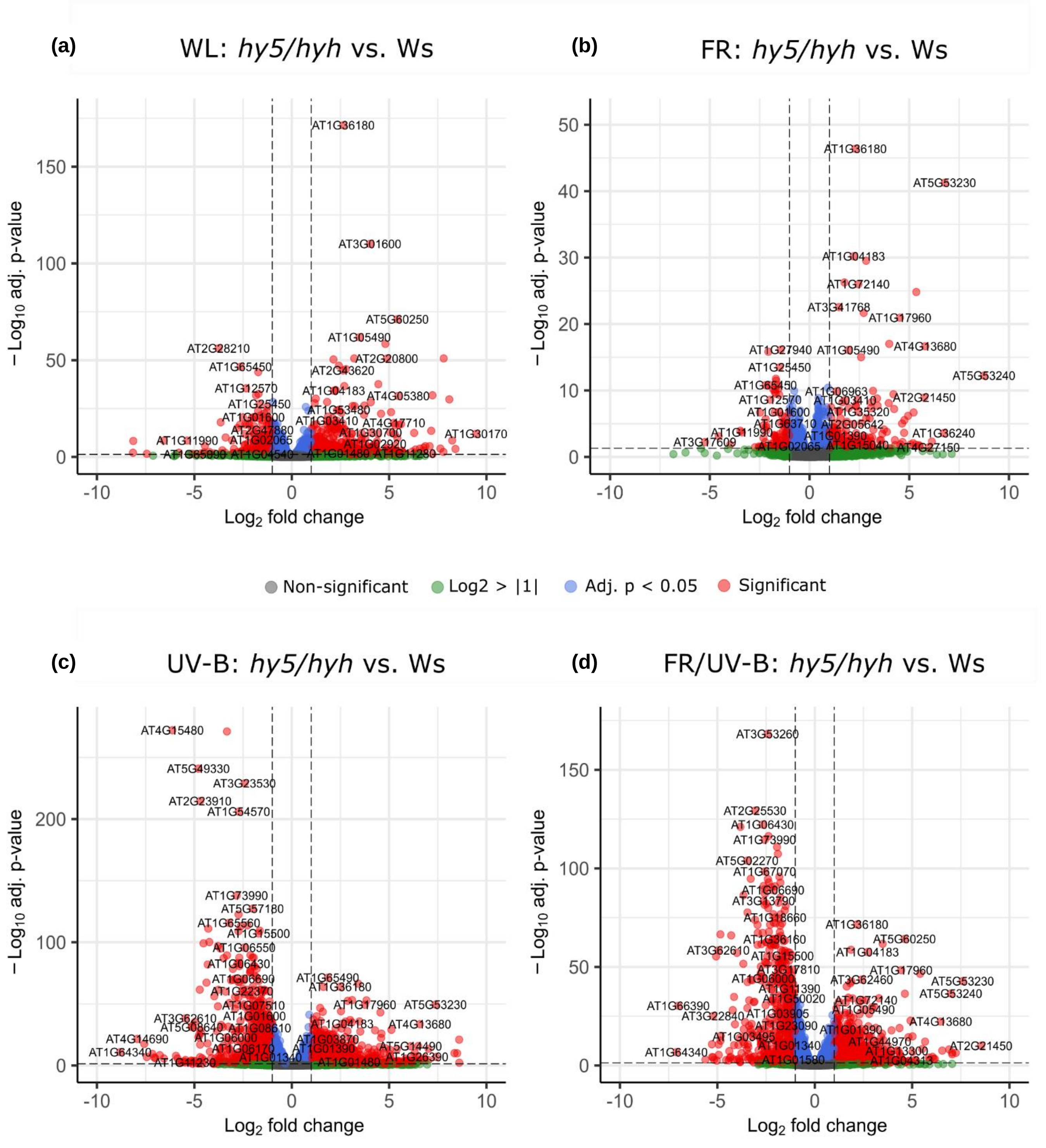

Figure S2. Volcano plots of differentially expressed transcripts in Ws and *hy5/hyh* seedlings treated with (a) WL, (b) WL+UV-B, (c) WL+ FR and (d) WL+ FR+ UV-B. Seedlings were grown for 7 d in WL at 20ºC before transfer at dawn to different light conditions (WL, +FR, +UV-B or +FR+UV-B) for 4 h. R:FR values were 8 (WL) or 0.06 (+FR). UV-B was provided at 1 $\mu molm^{-2}s^{-1}$.The x-axis represents log2 of the fold change in transcript abundance and the y-axis represents log10 of the p-value. Genes displaying significant fold changes > 2 (FDR corrected p value of <0.05) are shown in red. Transcripts induced by HY5/HYH (and therefore down-regulated in the *hy5/hyh* mutant) are shown on the left of each plot. Transcripts repressed by HY5/HYH (and therefore up-regulated in the *hy5/hyh* mutant) are shown on the right of each plot.

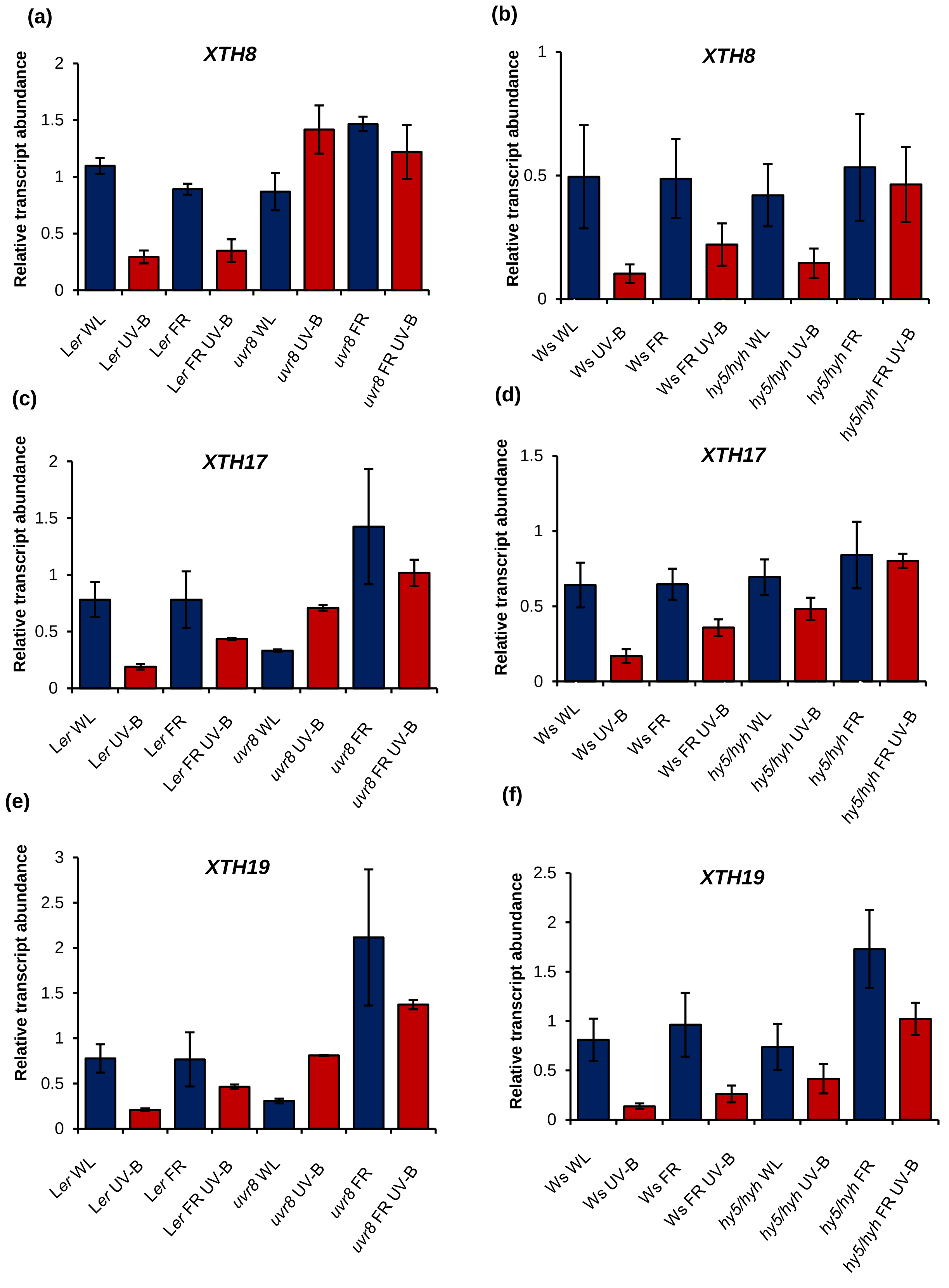

Figure S3. UV-B-mediated suppression of *XTH* transcript abundance requires UVR8. Relative abundance of *XTH8*, *XTH17* and *XTH19* transcript in L*er* and *uvr8-1* **(a, c, e)** and Ws and *hy5/hyh* (**b, d, f)** seedlings in different light conditions, measured by qPCR. Plants were grown for 10 d in 16 h light/ 8 h dark cycles at 20ºC before before transfer at dawn to WL, WL+UV-B (UV-B), WL+FR (FR) or WL+FR+UV-B (FRUV-B) for 4 h. R:FR values were 2.5 (WL) or 0.06 (+FR). UV-B was provided at 1 μmolm^{-2}s^{-1}. Data represent the mean of 2 (a, c, e) or 3 (b, d, f) independent biological repeats ± SE.

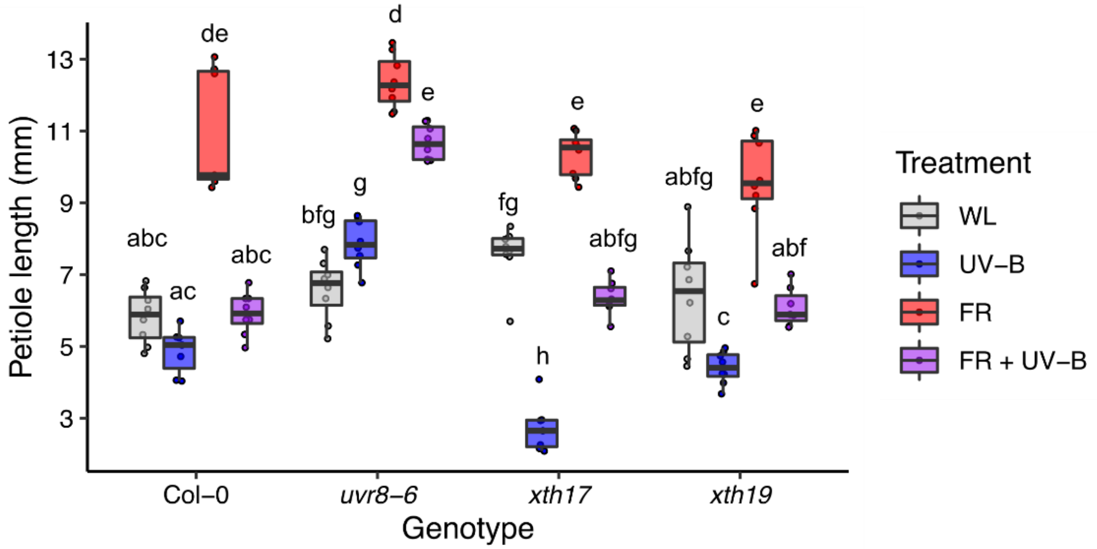

Figure S4. XTH enzymes are likely to act redundantly to control petiole elongation during shade avoidance. Plants were grown for 10 d in 16 h light/ 8 h dark cycles of WL before transfer to different light conditions: WL, WL+UV-B (UV-B), WL+FR (FR) or WL+FR+UV-B (FRUV-B) for 9 d. R:FR values were 2.5 (WL) or 0.06 (FR). UV-B was provided at 1 $\mu molm^{-2}s^{-1}$. The largest rosette leaf was used for petiole measurements. Boxes represent 25th to 75th percentile. Bars show the median petiole length of at least 7 plants in each treatment. Boxes represent 25th to 75th percentile, whiskers represent spread of data within 1.5 * interquartile range. ($n \geq 12$). Different letters represent statistically different mean values ($P< 0.05$) using a 2-way ANOVA with Tukey multiple comparison test.

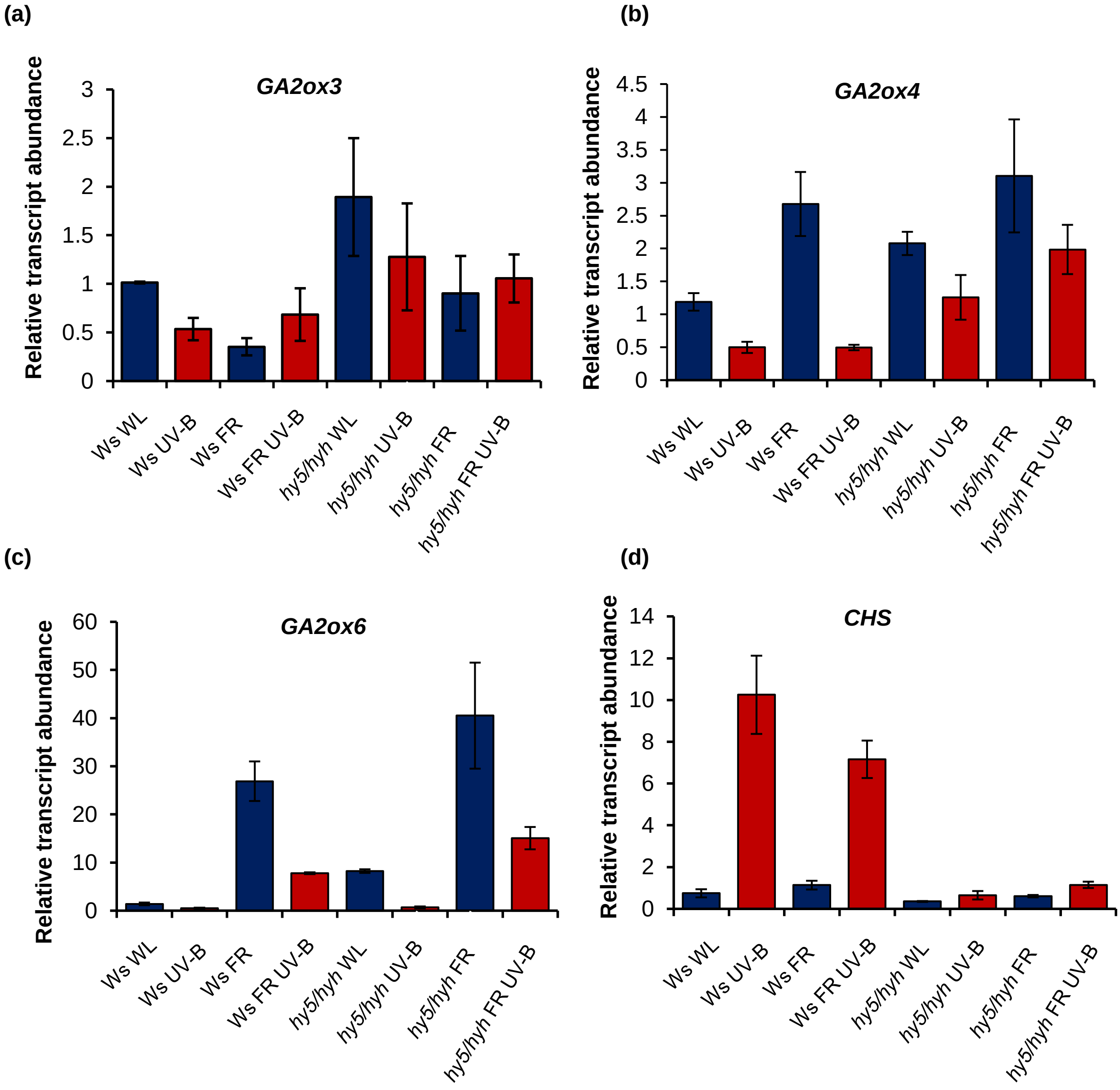

Figure S5. UV-B does not increase *GA2ox3*, *GA2ox4* or *GA2ox6* transcript abundance. Relative transcript abundance of *GA2ox3*, *GA2ox4, GA2ox6 and CHALCONE SYNTHASE* (*CHS*) in Ws and *hy5/hyh* mutants in different light conditions. Plants were grown for 10 d in 16 h light/ 8 h dark cycles at 20°C before before transfer at dawn to WL, WL+UV-B (UV-B), WL+FR (FR) or WL+FR+UV-B (FRUV-B) for 4 h. R:FR values were 2.5 (WL) or 0.06 (+FR). UV-B was provided at 1 μmolm^{-2}s^{-1}. Data represent the means of 2 (a-c) or 3 (d) independent biological repeats ± SE.

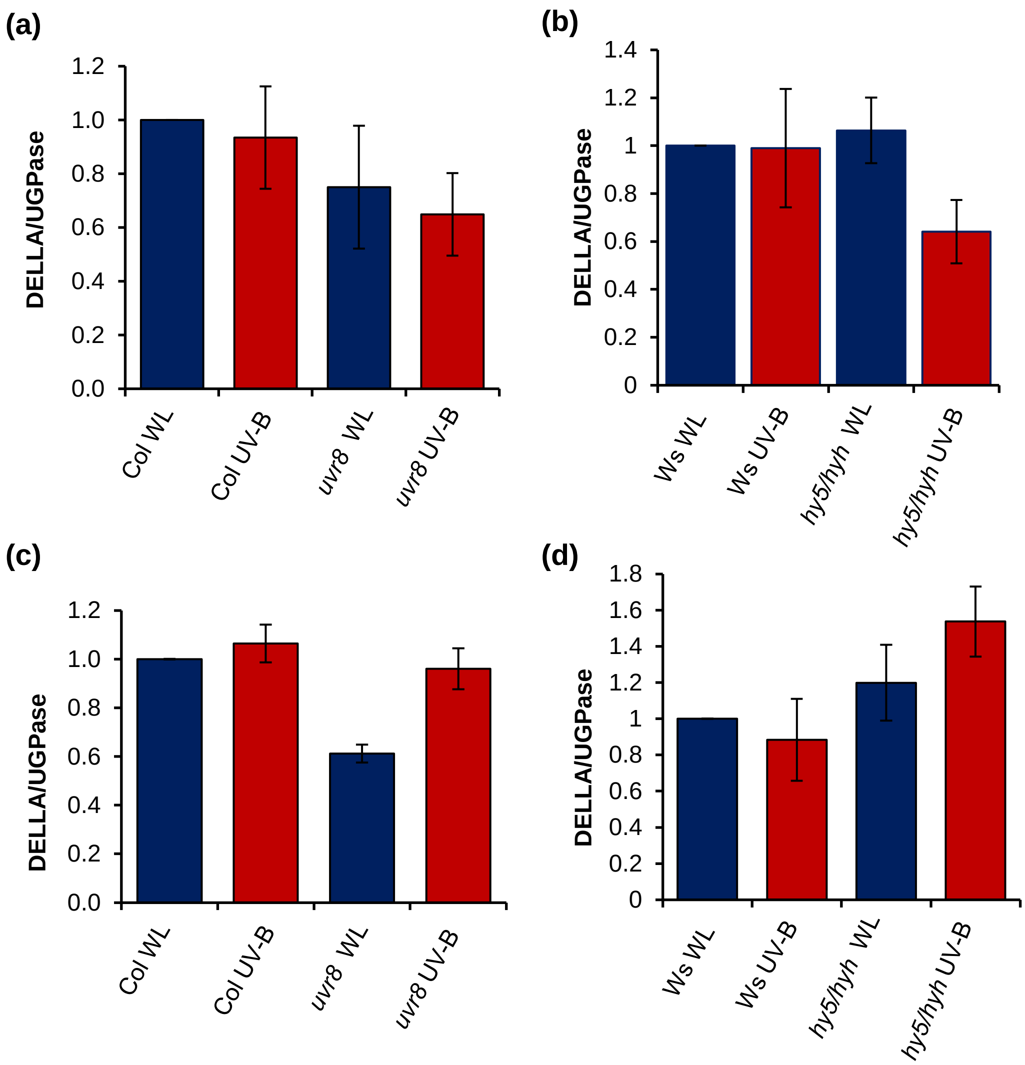

Figure S6. UVR8- and HY5/HYH- mediated increases in DELLA stability do not occur within 2 h of UV-B treatment. Quantification of RGA/UGPase ratios in Col-0 and *uvr8-7* (a,c) and Ws and *hy5/hyh* (b,d) using western blotting. Seedlings were grown for 10 d in 16 h light/ 8 h dark cycles at $20^{o}C$ before transfer at dawn to WL ± UV-B (1 $\mu molm^{-2}s^{-1}$) for for 1 h (a, b) or 2 h (c, d). Data represent the mean of 2 (a,b,c) or 4 (d) independent biological repeats ± SE.

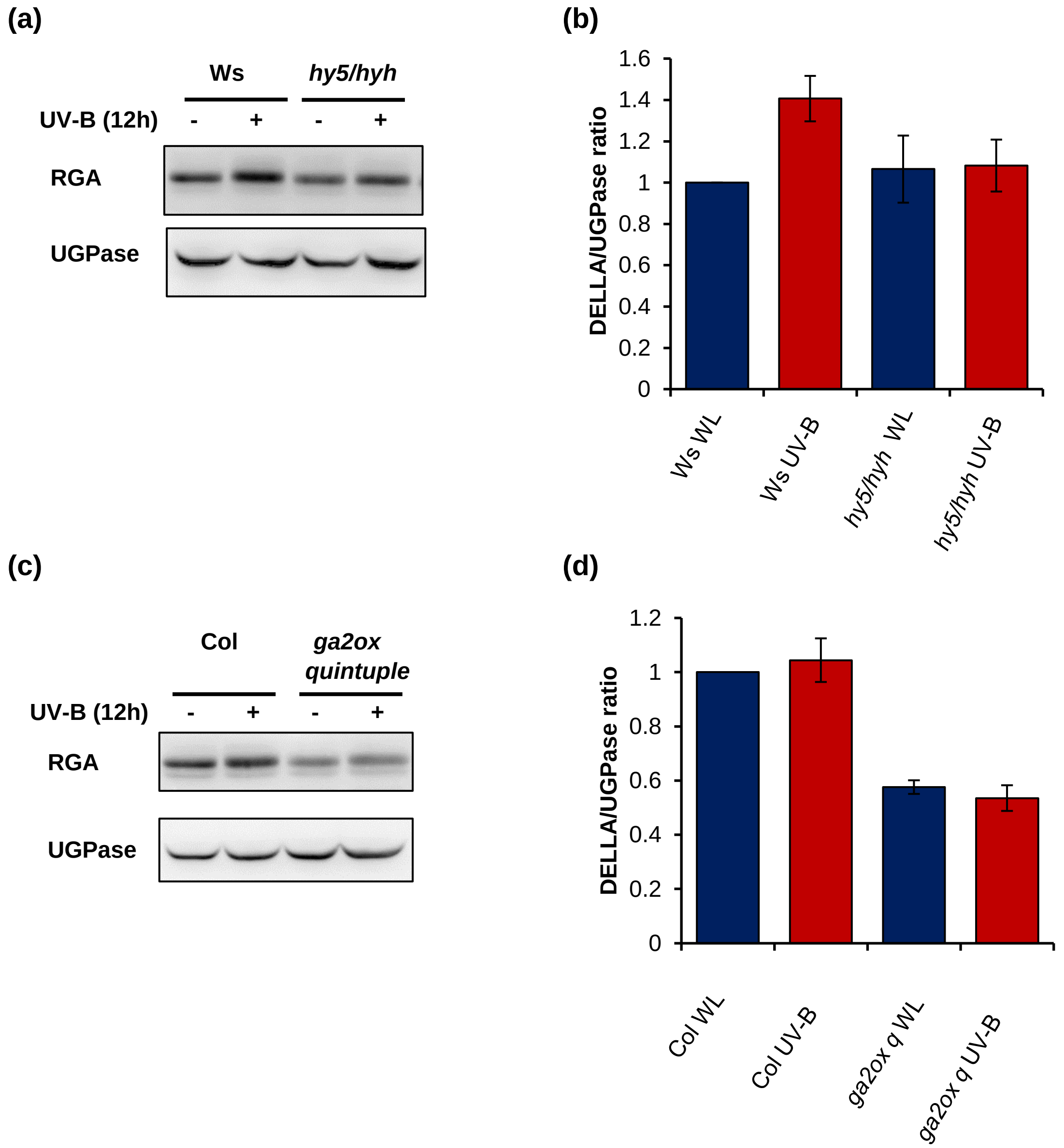

Figure S7. UV-B-mediated increases in DELLA stability involving HY5/HYH and GA2oxidases are maintained at 12 h in Ws but not Col-0. (a, b) Western blots of the DELLA protein, RGA and a UGPase loading control in **(a)** Ws and *hy5/hyh* and (b) Col-0 and *ga2ox q*. Seedlings were grown for 10 d in 16 h light/ 8 h dark cycles at 20°C before transfer at dawn to WL ± UV-B (1 µmolm^{-2}s^{-1}) for 12 h. **(b, d)** Mean DELLA/UGPase ratios of 3 **(b)** and 5 **(d)** independent biological repeats ± SE.

**Primer name**	**Sequence**
xth17 SALK_015077 _LP	TGAAAGAATTTAGTGTTCTTACTGGG
xth17 SALK_015077 _RP	AAAATCTCCGGGAACTACGTG
xth19 SALK_034274_LP	CTCCAGTTTTACCGTTGATGG
xth19 SALK_034274_RP	CGAGTTACGACCAAACAGACC
GA2ox2 F	CTGCGAGGAGTTCGGGTTCTT
GA2ox2 R	TTTTTGTCGACCCTCCACACC

Table S8. Primers used for genotyping *xth* and *ga2ox2* mutant lines.

**Primer name**	**Sequence**
GA2ox2 F	TAATCCTCAGCTCTCCTCTCCTAAA
GA2ox2 R	TCTCAGCATTTTACTCAGAGTGTCC
GA2ox3 F	AGGAGAAGCTGAGCCGTTT
GA2ox3 R	TTCTCCGGGTAATGGTTCAT
GA2ox4 F	GGCTCCAAGTGTCCAATTCA
GA2ox4 R	TCCTACATTGACGCAGAAAGC
GA2ox6 F	GGGACAGAAGTCTAGCGAAGTG
GA2ox6 R	TCGCTACGAACGTCTCTGATC
XTH8 F	TCT CTTCTTACAAGGACTTCGCC
XTH8 R	TGATCCCACCAATTCTCTGTTGT
XTH17 F	ACGTTCAGATACATTGGGGTGAT
XTH17 R	TTTGTCGAGCGAGAGAGAAAGAA
XTH19 F	CGGGAACTACATGGGATGAGATT
XTH19 R	CTTTGTCTCCTGAGCCTTTTGTG
ACTIN2 F	TCAGATGCCCAGAAGTGTTGTTCC
ACTIN2 R	CCGTACAGATCCTTCCTGATATCC

Table S9. Primer sequences used for qPCR. F = forward primer, R = reverse primer.
